# Supplementary material for: Dietary AhR Ligands Have No Anti-Fibrotic Properties in TGF-β1-Stimulated Human Colonic Fibroblasts
Source: Nutrients. 2022 Aug 9;14(16):3253. doi: 10.3390/nu14163253 (PMC9412321; doi:10.3390/nu14163253)
Supplement: Supplementary file 1 [file nutrients-14-03253-s001.zip › nutrients-1813133-supplementary.pdf]

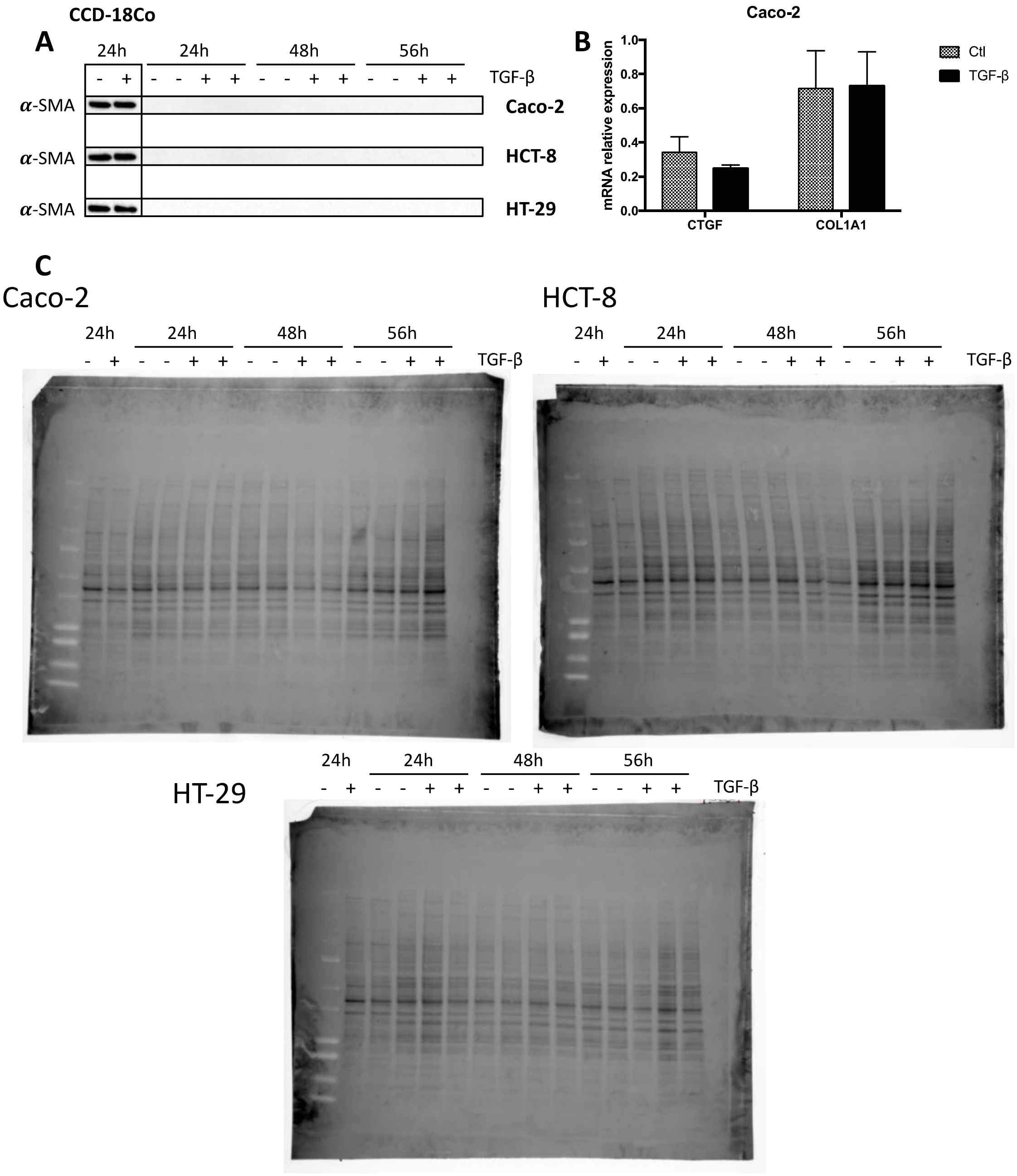

A: αSMA expression in CCD18Co (positive control) , Caco-2, HCT-8 and HT-29 in response to TGFβ for 24, 48 and 56h.

B: CTGF and COL1A1 mRNA levels in Caco-2 cells in response to TGFβ .

C: Total proteins gels from CCD18-CO, Caco-2, HCT-8 and HT-29 samples used in panel A.
